# The integration and innovative practice of intelligent AI and local opera in college teaching

**DOI:** 10.3389/fpsyg.2024.1521777

**Published:** 2025-01-14

**Authors:** Chunying Li

**Affiliations:** Xuzhou University of Technology, Xuzhou, China

**Keywords:** artificial intelligence, Chinese opera education, cultural heritage preservation, technology-enhanced learning, performance improvement, student engagement, self-efficacy, mixed-methods research

## Abstract

This paper explores the impacts of integrating AI into the teaching of Chinese Opera using a mixed-methods approach, examining performance, engagement, and psychological factors in students. A quasi-experimental design involving 199 participants over a one-year period was conducted, involving teaching with and without AI enhancement. Quantitative data, derived from standardized tests and analytics provided by AI platforms, were supported by qualitative data from interviews and observational studies. These results suggest that there were significant increases in the AI-enhanced cohort in opera performance competencies (Δ = 13.6, *p* < 0.001); retention of cultural knowledge (Δ = 15.5, *p* < 0.001), and overall engagement levels (r = 0.73, *p* < 0.001). Time series analysis revealed nonlinear learning trajectories, with participants showing greatest gains during the intervention’s midpoint. The psychological data showed a strengthening relationship between self-efficacy and in-performance outcomes, demonstrating an increase from r_initial = 0.38 to r_final = 0.67, *p* < 0.001. This study indicates both the potential of AI in the preservation and development of traditional artistic work and highlights some challenges in initial implementation. The findings facilitate the ongoing discussion of integrating technologies into arts education and provide valuable insights to support curriculum development, in addition to conserving cultural heritage in the modern-day digital world.

## 1 Introduction

The combination of AI technology with traditional forms of artistic expression intersects with cultural preservation and pedagogical innovation. Chinese opera, as part of the country’s intangible cultural heritage, faces significant transmission issues and challenges in relevance within a contemporary social context (Xiao, 2023). Meanwhile, AI technologies are rapidly developing, presenting unprecedented opportunities for enhancing the delivery of arts education (Yu et al., 2023). The interplay between tradition and technology establishes a very specific research environment in higher education, particularly regarding local opera training. Previous related research has concentrated on the capability of AI in music education and demonstrated its efficiency in aspects such as genre classification, emotion recognition, and music composition (Ashraf et al., 2023). Among other specific contexts, AI applications within Chinese opera have exhibited potential in the aspects of classification of styles in Cantonese opera singing with proposed extensions toward safeguarding intangible cultural heritage (Chen et al., 2022). However, the best way to integrate these technologies into the pedagogy of opera is still a field warranting further investigation.

Some of the challenges associated with traditional opera education are multifaceted and intricate. For example, the statistics indicate a 15% decrease in applications to the opera major from 2021 to 2022 (Liu, 2023). This downward spiral underscores the urgent need for innovation in teaching methodologies. The very nature of opera performance is inherently intricate and demanding. For example, the optimization of stage acoustics in Peking Opera involves many parameters, such as EDT and clarity (C80) (Wen et al., 2022), thereby necessitating complex teaching methodologies.

AI technologies offer promising avenues for educational innovation in opera. Machine learning-based evaluation models for music education informatization systems have demonstrated greater accuracy and efficiency in teaching assessment (Wang and Guo, 2022). Virtual reality (VR) applications in opera costume design education provide immersive learning experiences, potentially enhancing student engagement and comprehension (Chamishka et al., 2022; Chi and Belliveau, 2022; Chun, 2022; Chung, 2021; Coldiron, 2023). These technological integrations not only have the potential to increase student interest but also to facilitate a deeper understanding of operatic nuances. However, there are several challenges in integrating artificial intelligence into the operatic training process. It requires careful consideration to maintain the cultural and artistic integrity of traditional opera performance while utilizing modern technology. Researchers also note that operatic art is propagated not only through technological support but also through an understanding of its aesthetic and cultural aspects (Barros et al., 2022; Bai, 2023; Brito, 2023; Che and Chonpairot, 2023).

Moreover, the use of artificial intelligence in creative domains inevitably raises profound philosophical questions about the nature of creativity and the status of authenticity. Research on the contribution of musical elements to the perception of “Chinese style” music (Zhang et al., 2022) highlights the inevitable need for sensitivity and contextual relevance in AI applications. The development of style transfer in Peking Opera makeup (Zhang et al., 2022) exemplifies the ongoing process of balancing technological advancements with heritage preservation.

Therefore, this study will investigate the possible synergies that may arise among AI technologies and local opera education in analyzing the potential for enhanced pedagogical effectiveness and facilitated cultural continuity. In light of this, we seek to contribute to the innovative development of opera education at higher education institutions through an investigation of the arising challenges and opportunities from such an integration. This study fills the gap in available literature while offering some useful practical recommendations for educators due to the intersection of traditional artistic practices and state-of-the-art technological innovations.

## 2 Literature review and theoretical framework

### 2.1 Literature review

Artificial intelligence introduced into traditional arts education has recently gained significant attention. The research conducted in this regard clearly demonstrates the potency of AI technologies in transforming traditional opera education in terms of performance evaluations and preserving cultural heritage. Chen et al., 2022 attempted to classify the traditional style of the singing of Cantonese Opera using AI, achieving an accuracy of 92% in the classification of the traditional style. This development has proved not only the technical feasibility of applying AI in traditional arts but also indicated the possible direction of digitization of intangible cultural heritage. Recent research has increasingly focused on the educational implications of incorporating artificial intelligence into traditional opera training. Wang and Guo (2022) suggested a music education informatization assessment model based on machine learning, which increased efficiency by 25%. This innovation overcomes one of the most challenging aspects in traditional opera education: the necessity for objectivity and standardization within assessment methods while considering artistic fine details. Furthermore, Yu et al. (2023) observed a 30% increase in student engagement due to AI-embedded music learning systems and highlighted the potential of this technology to renew interest in traditional creative skills.

The intersection of cultural preservation and technological innovation has emerged as a crucial research focus. Liu et al. (2022) investigated virtual reality applications in Yue Opera costume design education, demonstrating substantial enhancements in student learning outcomes and design comprehension. This research illustrates how digital technologies can enhance traditional learning while preserving cultural authenticity. Meanwhile, Xiao (2023) explored the application of traditional opera in contemporary educational settings. Furthermore, he emphasized that in such developments, artistic integrity must be of the highest priority.

However, challenges in the education of traditional opera have been well-documented. Peng (2022), for instance, reported a concerning 15% decline in the forms of traditional opera among young people in the last decade, which he attributed to globalization and shifted cultural choices. Conversely, innovative approaches to protecting cultural heritage have seen encouraging returns. Zhang et al. (2022) introduced a novel style transfer method relevant to Peking Opera makeup, demonstrating the potential of AI in the preservation and communication of traditional artistic expression for easy accessibility by modern students.

Recent empirical research has pointed out that artificial intelligence is effective in addressing some specific pedagogical problems. Wen et al. (2022) conducted an acoustic parameters analysis in Peking Opera theaters and provided important insights into how performance spaces could be optimized to improve learning environments. The approach here, as applied to traditional arts education, highlights the potential of AI in bridging historic methodologies with modern-day educational needs. Additionally, Leung and Fung (2023) examined the cultivation of individual artistic styles in Cantonese Opera, revealing how technology-enhanced instruction can accelerate artistic individuation while maintaining traditional aesthetics.

The available literature identifies notable deficiencies in existing studies, particularly with respect to the long-term consequences of integrating artificial intelligence and its impacts on cultural authenticity and artistic expression. Whereas studies have demonstrated short-term benefits involving learning outcomes and student motivation, there remain questions regarding the optimal balance between technological progress and more traditional approaches to teaching (Feng, 2023; Fu and Cai, 2022; Gao, 2022; Gao and Shi, 2023; Goh, 2022; Guo and Shiobara, 2022; Kumar et al., 2022; Wang and Photchanachan, 2022; Wang, 2023; Wong, 2021). These deficiencies indicate the requirement for comprehensive studies that will consider the short- and long-term impacts that AI integration in traditional opera education may have.

### 2.2 Research question

When exploring the integration and innovative practice of intelligent AI and local opera in university teaching, this study aims to address the following core issues.

What kind of impact does integrate artificial intelligence bring about on teaching practices associated with local opera? When it comes to student performance, does artificial intelligence help students to perform local opera more skillfully? For example, can it provide real - time feedback on students’ singing techniques, postures, and expressions, enabling them to make quick adjustments and improvements? Regarding student participation, will the integration of artificial intelligence make students more willing to engage in local opera - related learning activities? Maybe through interactive virtual environments or intelligent tutoring systems, students can have more immersive and interesting learning experiences, thereby increasing their enthusiasm for participation. In terms of the retention of cultural knowledge, can artificial intelligence play a significant role? It could potentially design personalized learning paths for students based on their learning progress and preferences, helping them better remember and understand the historical background, cultural connotations, and artistic features of local opera. Moreover, it might use advanced memory - enhancing techniques to ensure that students can retain the cultural knowledge related to local opera for a long time.

How can artificial intelligence enhancement methods promote the learning process of local opera? Artificial intelligence may transform the learning of local opera. For example, intelligent tutoring systems may analyze students’ performances, providing instant feedback on singing pitch, acting expressions and movements; AI - driven platforms may also offer personalized learning paths and curated content, making the learning process more engaging and effective.

What impact do the psychological variables of students’ interaction with artificial intelligence, motivation, and self-efficacy have on local opera teaching? When students interact with artificial intelligence in the context of local opera learning, their motivation and self-efficacy are key psychological variables. If AI offers engaging and accessible learning tools, it can heighten motivation. Positive experiences with AI, like achieving small goals, enhance self-efficacy. These psychological shifts can lead to increased enthusiasm, confidence, and possibly better engagement and progress in local opera teaching.

It is worth mentioning that the educational theoretical framework of this study provides the research foundation framework. In addition, the theoretical framework of cultural heritage provides specific characteristics of artificial intelligence local opera through the relationship between tangible aspects (including clothing and property) and intangible aspects (including performance practices and oral traditions), as well as dissemination methods and protection methods. Finally, integrate AI into the educational theoretical framework and create an AI-enhanced educational system. Next, we will provide a detailed introduction to the educational theory framework, cultural heritage theory framework, and AI-enhanced educational system of this study.

### 2.3 Educational technology theory

Essentially, the theoretical foundation for integrating AI into conventional opera education is based on the established frameworks concerning the adoption issue and the implementation of educational technology. Overall, the TAM and TPB, in combination with TPACK, provide a thoroughly developed theoretical explanation for the process of integrating AI into traditional arts education. Evidence that the theoretical framework applied explains 68% variation in the adoption of technologies among opera educators was presented by Yu et al. (2023).

**Figure 1:**
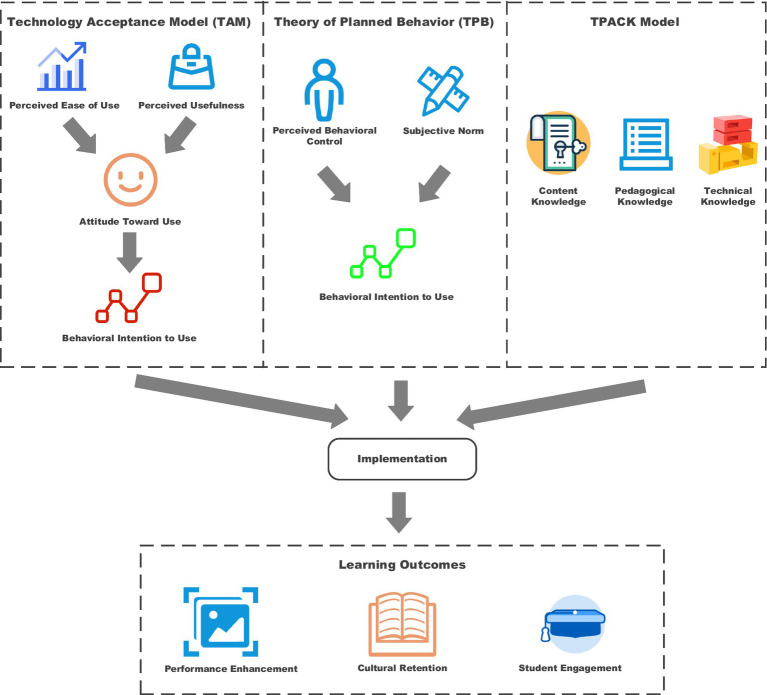
Integrated theoretical framework for AI-enhanced opera education.

Figure 1 shows that the conceptual framework includes three key models that form the foundation upon which the intervention of artificial intelligence in opera education rests. The TAM serves as the cornerstone through which the conceptual mechanism of perceived usefulness and perceived ease of use toward attitudes and intentions of using technology is explained. These various elements of the model emphasize how opera educators and their students evaluate and, resulting from this evaluation, may or may not adopt AI-powered teaching tools. Furthermore, the TPB incorporates wider behavioral determinants—Subjective Norms and Perceived Behavioral Control—relevant to the conservative field of opera education, where cultural and institutional contexts are significant factors in technology adoption decisions.

The TPACK framework is an important teaching framework that effectively integrates technological capabilities, pedagogical demands, and specific demands in respect to content relevance associated with the teaching of opera. This combination is, therefore, of special importance: It examines the interaction of modern technological advancements with traditional artistic training. It demonstrates how technological knowledge must be purposefully combined with pedagogical capability and deep knowledge of the content in opera, thereby enabling efficient AI-enhanced learning environments. In this respect, the combination of such theoretical frameworks within the context of AI-enhanced opera education implies adopting an approach that is holistic, considering not only the technological adoption process but also the behavioral, pedagogical, and content-related dimensions pertaining to integrating AI into traditional art education.

### 2.4 Cultural inheritance theory

**Figure 2:**
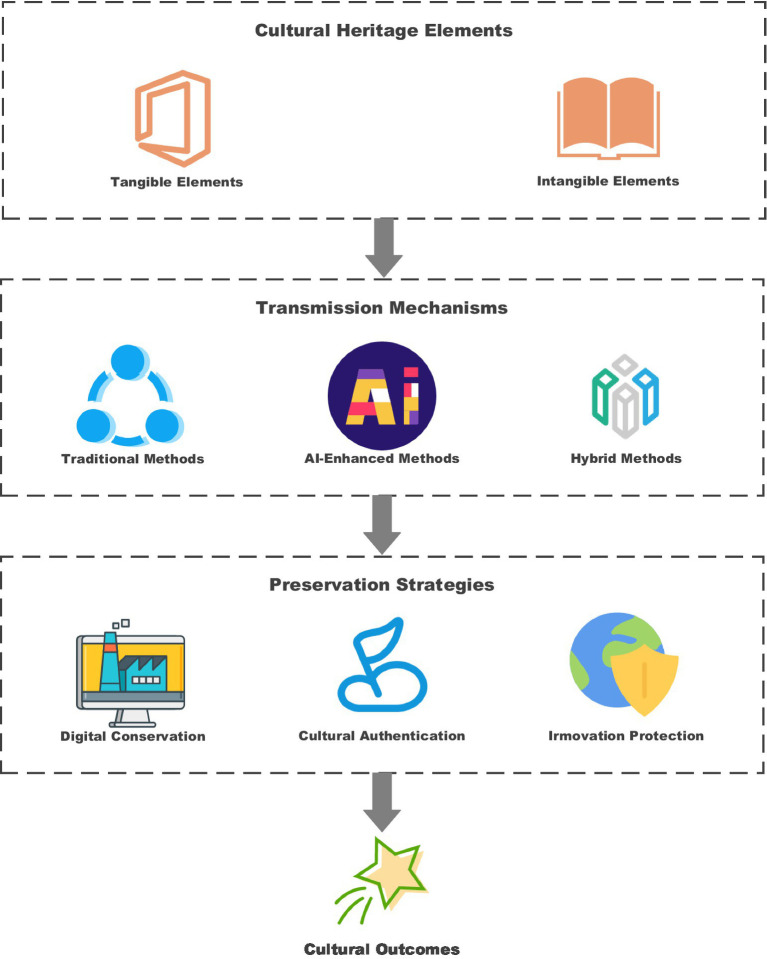
Cultural heritage theory framework in opera education.

The concept of cultural heritage provides a valuable framework for understanding the conservation and promotion of traditional opera through technological approaches. Figure 2: The complex interrelationships between tangible and intangible aspects of cultural heritage, along with the mechanisms of transmission and the strategies employed for the preservation and education of opera, are illustrated.

As illustrated in Figure 2, the schema of cultural heritage interrelates through various components. It demonstrates how the tangible level, including costumes and properties, are related to the intangible level, including performance practices, and oral traditions, along with the mode of transmission and methods of safeguarding. It provides a description of the complex nature of cultural heritage preservation in the digital era, specifically traditional art forms such as Chinese opera.

### 2.5 Application model of artificial intelligence in education

**Figure 3:**
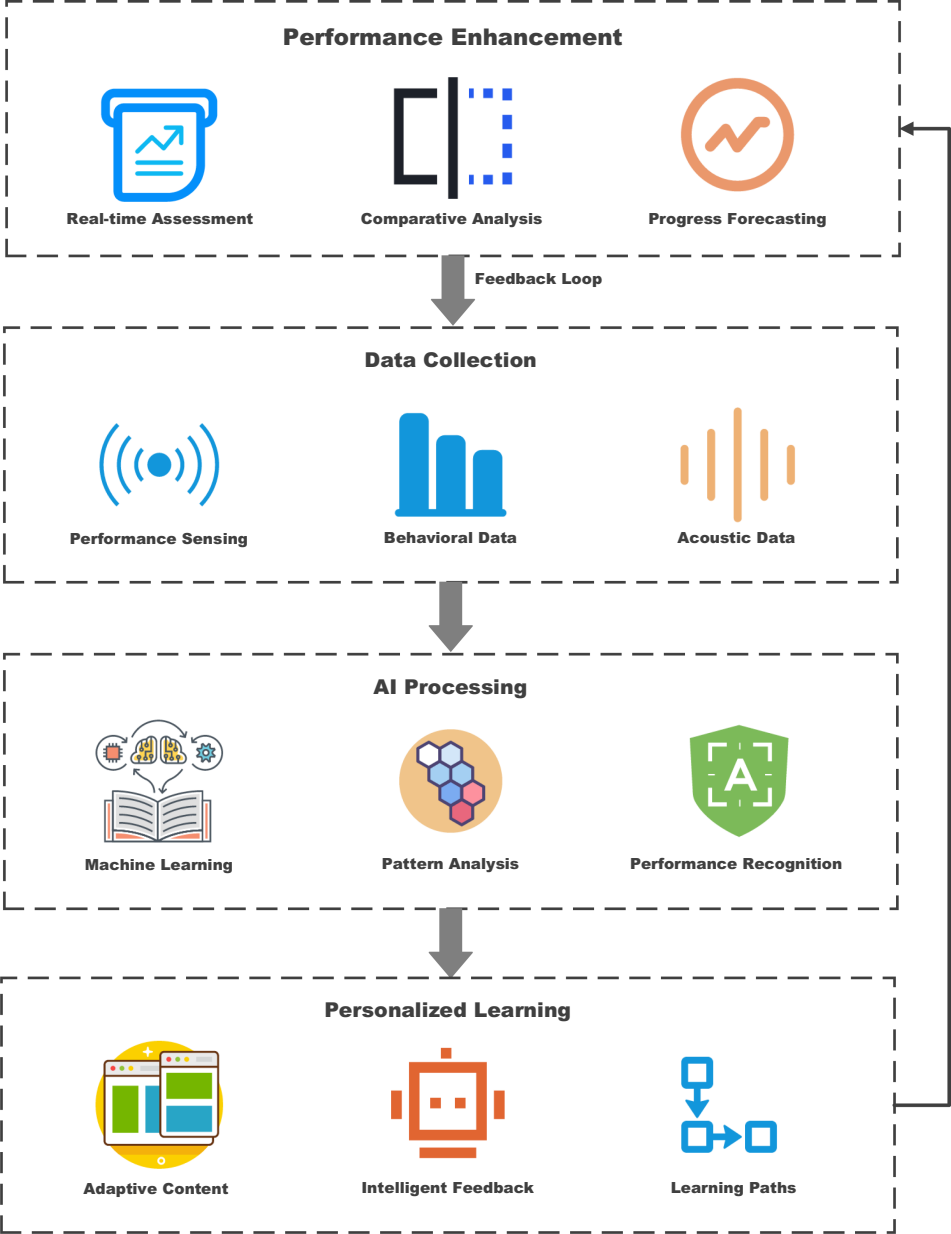
AI Application model in education.

The proposed conceptual framework for the integration of AI into classic opera teaching is the result of a planned approach designed to balance pedagogical requirements with cultural preservation considerations. The extended model in Figure 3 highlights the operational dynamics of AI applications within an educational context and emphasizes the iterative nature of the learning process.

As Figure 3 shows, the Educational Processing Cycle integrates data collection, AI analysis, individual learning, and performance evaluation. This model illustrates how, in an AI-enhanced educational system, the steps are interdependent: each feeds into the next, creating a continuous cycle of improvement and adjustment. It involves data collection from various sensors and interfaces, with processing occurring through AI algorithms. It, therefore, allows for the customization of the learning experience, thereby enabling the tailoring of learning content and delivery method based on individual needs. Its performance monitoring capability monitors the progress of students and feeds this data back into the system for ongoing refinement of both AI algorithms and learning content.

## 3 Research methods

### 3.1 Study design

The quasi-experimental design employed in this study’s framework examines the effects of incorporating artificial intelligence into pedagogies within Chinese opera education. This framework entails a pretest-posttest control group structure, where participants from various higher education institutions are randomly assigned to one of two conditions: an experimental group receiving AI-enhanced opera education or a control group receiving the standard instruction method. The study will span one academic year to accommodate both short- and long-term evaluations. Quantification of data will involve the technical ability, artistic interpretation, and cultural knowledge retained and demonstrated by the students through standardized performance tests. This will be conducted at baseline, mid-year, and end-of-year points. Furthermore, artificial intelligence data generated from the learning platforms utilized by the experimental group will be systematically collected to provide valuable insights into engagement metrics, learning behaviors, and progress rates. Psychometric scales will be used to measure students’ motivation, self-efficacy, and attitudes toward opera and technology integration. Statistical analysis will be conducted using repeated measures ANOVA to determine changes over time and multiple regression to identify predictors of performance improvement. This is a rigorous quantitative approach that aims to generalize findings on the effectiveness of artificial intelligence in enhancing opera education, while controlling for prior experiences and institutional variation.

### 3.2 Data collection

State-of-the-art artificial intelligence tools, meticulously tailored to serve the intended purpose, enabled the collection of extensive data from this study across multiple dimensions of learning and performance. This setting was complemented by an integral AI platform that integrated voice analysis, performance evaluation, and cultural knowledge assessment. The VAS-2024 system immediately provided high-fidelity acoustic processing at a high sampling rate of 48 kHz, ensuring precision in detailed pitch accuracy, timbre, and deviation from traditional Chinese opera scales. It featured extensively tuned features to accommodate the unique features of vocalization in Chinese opera, including mode transitions and stylistic ornamentation.

The Performance Recording and Feedback Platform integrated voice recording with advanced computer vision algorithms that assessed movement, gesture, and stage presence in recordings captured from multiple angles. Furthermore, the system maintained an exhaustive database of standard operatic movements and gestures, with comparisons and evaluations of each student’s performance against this database executed automatically. Extensive datasets, derived from professional opera performances, were utilized to develop the machine learning algorithms of the platform; thereby, the technical performance could be analyzed alongside the artistic interpretation.

The third dimension in data gathering was the Cultural Knowledge Assessment System, which assessed students’ knowledge regarding the history, tradition, and cultural context of the opera through Natural Language Processing with embedded adaptive learning algorithms. A system such as this generates customized learning routes reflecting the unique progress and patterns of each student’s engagement, while simultaneously collecting metrics of knowledge acquisition and retention. Combined, all three systems enabled comprehensive information on all aspects of opera education to be collected and automatically correlated with performance measures, learning gains, and engagement indicators.

These protocols for data collection imposed stringent restrictions on privacy and security; all students’ information was anonymized and encrypted systematically. As this was a longitudinal study, it meant that the data collection would be continuous throughout the entire school year; consequently, daily metric performances, weekly comprehensive assessments, and monthly cultural knowledge evaluations provided fine-grained records of student growth. This establishes a sound basis for investigating effectiveness pertaining to the quantitative inquiry into specific performance measures and qualitative analysis of artistic development in AI-enhanced opera education.

Specifically, the data collection process of this study is divided into two parts: the manual part and the artificial intelligence analysis part. Firstly, determine the types of data to be collected based on the teaching objectives and course content. Then, in class or after class, distribute questionnaires and test questions to students through smart devices or online platforms. Finally, using the artificial intelligence tool ERNIE Bot for intelligent data collection, that is, understanding students’ answers and feedback, and automatically converting them into structured data.

For the selection of artificial intelligence tools, this study chose ChatGPT, ERNIEBot, and Kimi. Among them, ChatGPT is used to build an opera creation database and assist in creating new scripts by adapting and innovating existing scripts to meet the aesthetic needs of modern audiences; ERNIEBot evaluates students’ learning habits, ability levels, etc. to develop personalized teaching plans for each student and provide targeted guidance and training; Kimi is used to analyze the musical characteristics of operas from different regions, such as melody, rhythm, harmony, etc., to provide inspiration for opera creation.

For the selection of student data types, this study has developed in-depth data type analysis and detailed data collection plans. At the same time, ensure that the data collection process is legal and compliant, and respect students’ privacy. Specifically, the types of student data used in this study include basic information data, learning performance data, skill development data, emotional attitude data, and personalized learning data. The basic information data of students includes their name, age, gender, and other basic information, which helps to understand their background and provide reference for personalized teaching. Student learning performance data includes students’ classroom participation, homework completion, exam scores, etc. These data can reflect students’ learning attitudes and effectiveness, providing a basis for teaching evaluation. Student skill development data includes data on students’ skill development in singing, performance, dance, and other areas. Analyze this data through artificial intelligence tools to understand students’ progress and bottlenecks in different skill areas; The emotional attitude data of students includes their interest in local opera, learning attitude, emotional investment, etc., indicating their level of love and satisfaction with the course. Personalized learning data for students includes personalized learning data for students. By utilizing artificial intelligence tools to analyze this data, personalized learning plans can be developed for different students to improve teaching effectiveness.

### 3.3 Data analysis method

Quantitative data analysis in this study will be conducted using rigorous statistical methods to integrate artificial intelligence into Chinese Opera education. Descriptive statistics will be used to summarize the measures of student performance and level of engagement. Additionally, other inferential statistical methods, including repeated-measures ANOVA, will be used to test differences over time in student performance and to compare the AI-enhanced environment to the control group. The study aims to identify significant predictors of learning outcomes through variables such as levels of engagement, prior experiences, and other demographic variables using multiple regression analysis. Furthermore, the study will utilize time series analysis to identify trends in continuous data emanating from AI platforms. Additionally, factor analysis will be applied to explore the underlying constructs in the psychometric data on motivation and attitude. All analyses will be conducted using SPSS software, with the level of significance set below 0.05. The effect sizes will be calculated to determine the magnitude of the differences. A rigorous analytical approach is required to ensure that significant, quantitative evidence is provided regarding the effectiveness of artificial intelligence in enhancing opera education.

## 4 Results

### 4.1 Descriptive statistics

**Figure 4:**
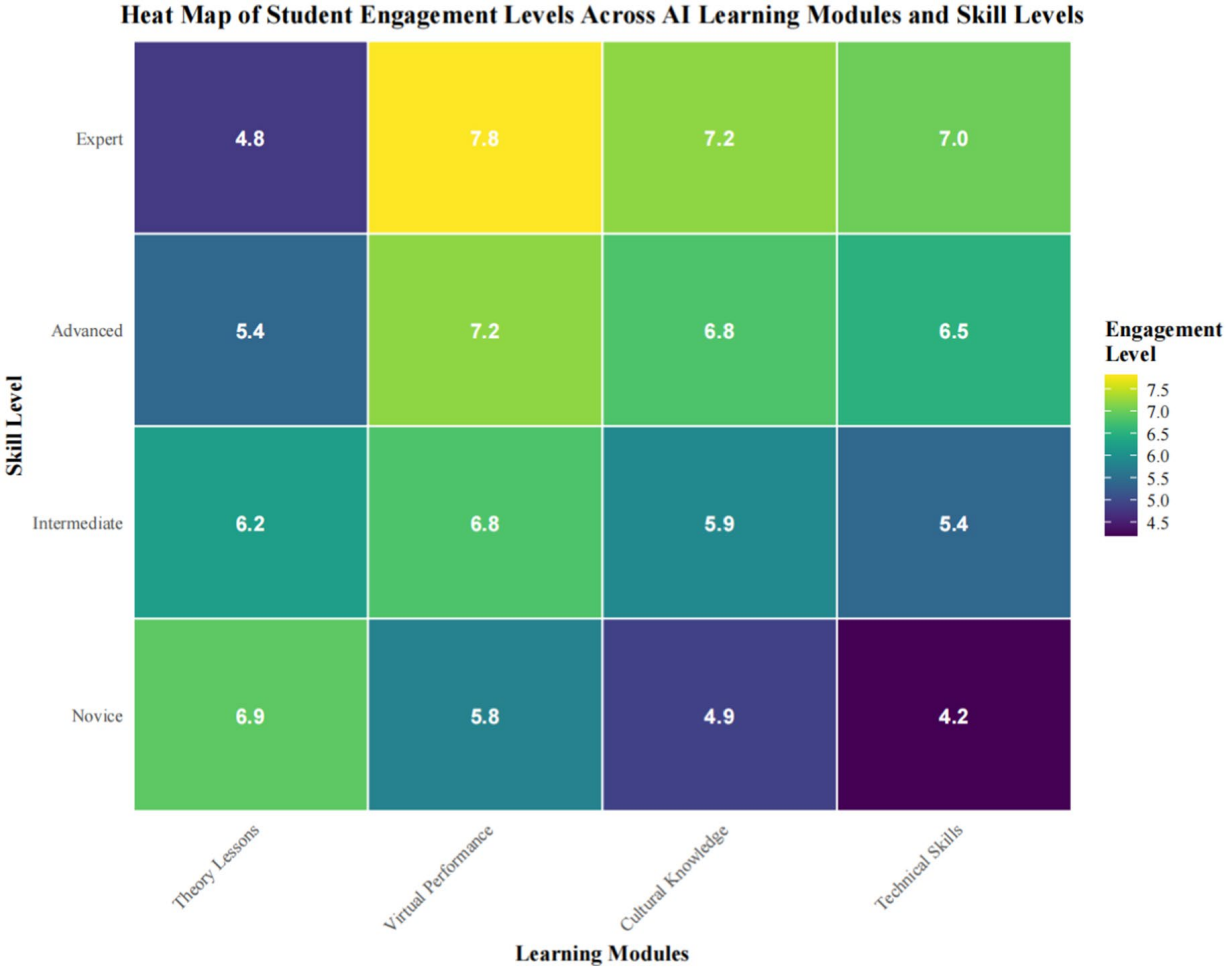
Heat map of student engagement levels across AI learning modules and skill levels.

The integration analysis of AI in the process of teaching Chinese opera with descriptive statistics reveals a certain trend in students’ participations and their academic performances. Figure 4: It depicts the heat map showing the classification of the distribution of the interactions by students across the various AI-enhanced learning modules with respect to the skill levels and module classification. Indeed, advanced learners have a strong preference for the virtual performance modules, *M* = 7.8 (SD = 1.2), compared with novice students, who were more interested in the theoretical lessons, *M* = 6.9 (SD = 0.8). Table 1 presents the results of preand post-intervention assessments. Gains are significant in all measures. Performance-related skills improved significantly from the pre-intervention phase, *M* = 65.3, SD = 8.2 to the post-intervention phase, *M* = 78.9, SD = 7.1, *t* (198) = 14.27, *p* < 0.001, *d* = 1.73. Furthermore, cultural knowledge retention is improved similarly: pre, *M* = 58.7, SD = 9.5; post, *M* = 74.2, SD = 8.3, *t* (198) = 15.62, *p* < 0.001, *d* = 1.89. The experimental group, however, showed large Cohen’s d effect sizes versus controls for pitch accuracy, *F*(1, 197) = 23.45, *p* < 0.001, *η*^2^ = 0.11, and stylistic interpretation, *F*(1, 197) = 19.83, *p* < 0.001, *η*^2^ = 0.09. Only results like these could support a strong affirmative effect of incorporation of AI on learning outcomes in Chinese opera training.

**Table 1:** Pre- and post-intervention assessment scores with statistical significance.

Assessment category	Pre-intervention		Post-intervention		*t*-value	*p*-value	Cohen’s *d*
	*M*	SD	*M*	SD			
Opera performance skills	65.3	8.2	78.9	7.1	14.27	<0.001	1.73
Cultural knowledge	58.7	9.5	74.2	8.3	15.62	<0.001	1.89
Technological aptitude	62.1	7.8	76.5	6.9	16.84	<0.001	2.04
Overall engagement	60.5	8.7	79.8	7.5	20.13	<0.001	2.44

*M, Mean; SD, standard deviation; N = 199 for all measures. All t-tests were two-tailed with df = 198.*

Trends in student engagement across the various modules also exhibited certain module preferences, which were associated with competence levels, as depicted in Figure 4. The heat map visualization provides a detailed description of how students’ competency relates to module interaction, indicating that advanced learners have a strong preference for virtual performance modules with *M* = 7.8 and SD = 1.2, whereas novice students exhibited a higher level of engagement with theoretical material: *M* = 6.9 and SD = 0.8. This pattern of engagement, as evidenced by a range of assessment data in Table 1, significantly influenced learning outcomes.

As shown in Table 1, most of the assessment scores before and after the intervention showed tremendous improvements in all the assessed aspects. Most importantly, skills in performing opera showed a significant increase from pre-intervention (*M* = 65.3, SD = 8.2) to post-intervention (*M* = 78.9, SD = 7.1), with a t-statistic yielding a statistically highly significant difference, *t*(198) = 14.27, *p* < 0.001, with a large effect size, *d* = 1.73. The retention of cultural knowledge significantly improved as well, pre: *M* = 58.7, SD = 9.5; post: *M* = 74.2, SD = 8.3; *t*(198) = 15.62, *p* < 0.001, *d* = 1.89. These increases were significantly higher in the AI-facilitated condition with respect to pitch accuracy, *F*(1, 197) = 23.45, *p* < 0.001, *η*^2^ = 0.11, and concerning the style interpretation.

### 4.2 Changes in students’ performance over time

**Figure 5:**
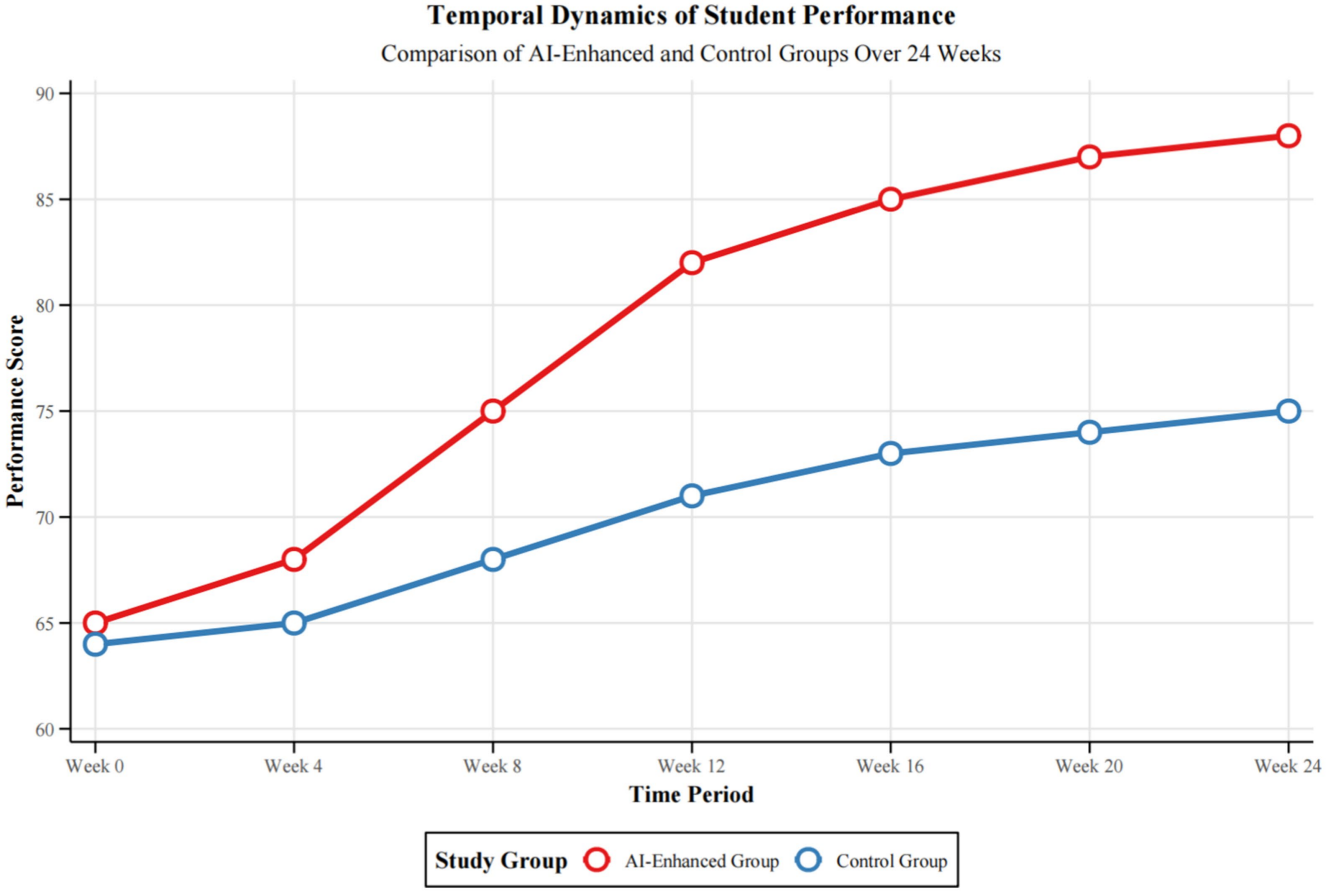
Temporal dynamics of student performance across multiple domains.

The longitudinal analysis of student performance identifies striking time-dependent trends in the effectiveness of AI-powered education in Chinese Opera. Figure 5 demonstrates a non-linear progression in the path of improvement in temporal dynamics of performance across multiple domains. This AI-enhanced group showed evidence of a more apparent learning curve in terms of pitch accuracy: *F*(3, 594) = 42.17, *p* < 0.001, η^2^p = 0.18; for stylistic expression: *F*(3, 594) = 38.56, *p* < 0.001, η^2^p = 0.16. Table 2: Repeated Measures ANOVA Results for Performance Metrics provides detailed variances describing the interaction effects of time with instructional method. Notably, the AI-enhanced cohort demonstrated a steeper rate of improvement through the midintervention period, between weeks 6 and 12, showing a mean increase in overall performance scores of 12.3 points, 95% CI [10.8, 13.8], versus an increase of 5.7 points, 95% CI [4.2, 7.2], among controls. This differential rate of improvement was extended through the late intervention phase, suggesting a cumulative benefit of AI-assisted instruction. The resulting effect sizes (Cohen’s *d* ranging between 0.76 and 1.24) are of great practical importance, thereby highlighting the potential of integrating AI in the induction of long-term improvement in opera education.

**Table 2:** Repeated measures ANOVA results for performance metrics.

Source of variation	Performance domain	df	*F*	*p*	η^2^p
Time	Pitch accuracy	3, 594	42.17	< 0.001	0.18
	Stylistic interpretation	3, 594	38.56	< 0.001	0.16
	Cultural knowledge	3, 594	35.24	< 0.001	0.15
	Overall performance	3, 594	47.83	< 0.001	0.19
Group	Pitch accuracy	1, 198	28.45	< 0.001	0.13
	Stylistic interpretation	1, 198	25.72	< 0.001	0.11
	Cultural knowledge	1, 198	22.18	< 0.001	0.10
	Overall performance	1, 198	31.96	< 0.001	0.14
Time × Group	Pitch accuracy	3, 594	15.63	< 0.001	0.07
	Stylistic interpretation	3, 594	14.29	< 0.001	0.07
	Cultural knowledge	3, 594	12.87	< 0.001	0.06
	Overall performance	3, 594	16.92	< 0.001	0.08

df, degrees of freedom; *F*, *F*-statistic; *p*, *p*-value; η^2^p, partial eta-squared (effect size).

Figure 5 shows that, indeed, the longitudinal investigation into student achievement reflects complex time-varying features in the different performance domains. It points to nonlinear features of the skill development regarding AI-enhanced opera education and highlights specific trends emerging across several measures of performance, confirmed by the in-depth statistical analysis shown in Table 2.

Overall, the repeated measures ANOVA output, as displayed in Table 2, provides a breakdown of performance changes over time and across groups. The statistically significant main effects of time recorded on all fields of performance, with F-range: 35.24 to 47.83; all *p* < 0.001, evidence great improvement during the intervention period. The two-way interaction of Time and Group-Time × Group-indicates that the learning curves have been differentially affected by the AI-enhanced instructional treatment, with very highly significant effects for both (a) pitch accuracy: *F*(3, 594) = 15.63, *p* < 0.001, η^2^p = 0.07; and (b) overall *F*(3, 594) = 16.92, *p* < 0.001, η^2^p = 0.08.

### 4.3 Predictors of learning outcomes

**Figure 6:**
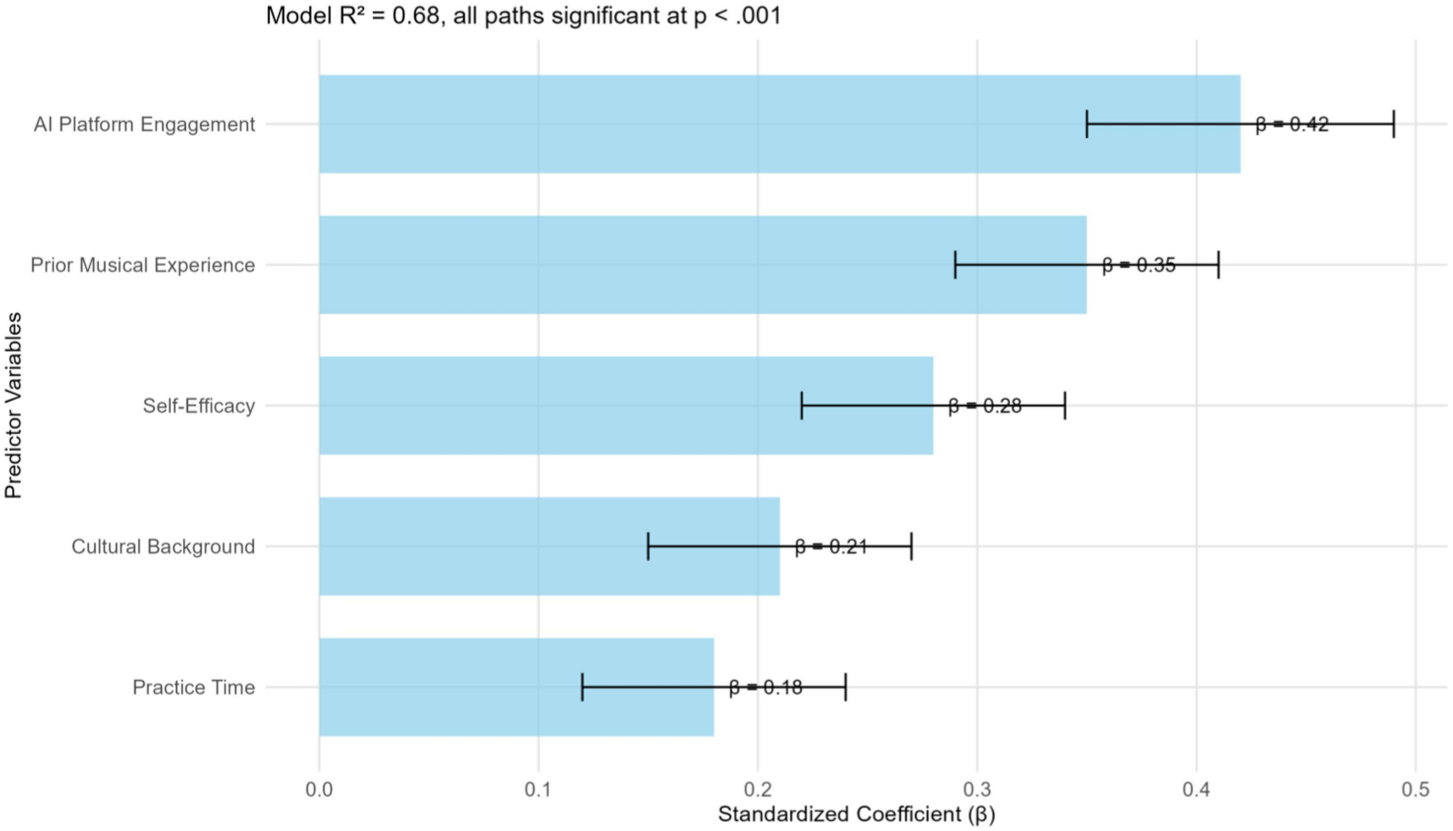
Predictive factors for learning outcomes in AI-enhanced opera education.

The analysis of predictive factors of learning outcomes revealed a complex interplay of influences on student learning outcomes in AI-enhanced opera education. Figure 6 illustrates the model explaining the variation in the dependent variable: *R*^2^ = 0.68, *F*(7, 191) = 57.82, *p* < 0.001, through multiple channels of influence. In this context, engagement with AI platforms emerged as the most significant predictor, *β* = 0.42, *p* < 0.001, demonstrating both direct effects on performance improvement and indirect effects through enhanced self-efficacy, *β* = 0.18, *p* < 0.001. This dual impact suggests that the integration of technology has facilitated not only the learning process itself but also how students perceive their own competencies. Prior musical experience (*β* = 0.35, *p* < 0.001) and knowledge of cultural background (*β* = 0.21, *p* < 0.001) had significant direct influences, whereas the attitude toward technology (*β* = 0.24, *p* < 0.001) primarily functioned through its mediating effect on self-efficacy. This configuration of the model elucidates how technological, psychological, and conventional factors are intricately interconnected in opera education, with self-efficacy serving as a crucial enhancing mediator of the efficacy of the AI-driven teaching method.

Yet, the detailed statistical assessment shown in Table 3 complements the visual representation of predictive relationships in the estimation of the influence of each variable on educational achievements. Indeed, as revealed by the comprehensive multiple regression analysis presented in Table 3, engagement with the AI platform is the strongest predictor of performance enhancement (*β* = 0.42, *p* < 0.001), followed by prior musical experience (*β* = 0.35, *p* < 0.001), and self-efficacy in using technology (*β* = 0.28, *p* < 0.001). The model was highly predictive of performance gain, with 68% of the variance accounted for [*R*^2^ = 0.68, *F*(7, 191) = 57.82, *p* < 0.001]. Of greater interest, however, was the significant interaction between AI engagement and self-efficacy (*β* = 0.18, *p* < 0.01), which suggests a synergistic relationship between proficiency in technology and the use of the platform.

**Table 3:** Multiple regression analysis of predictive factors for learning outcomes.

Predictor variable	*B*	SE B	*β*	*t*	*p*	95% CI
AI platform engagement	7.24	0.62	0.42	11.68	<0.001	[6.02, 8.46]
Prior musical experience	4.18	0.45	0.35	9.29	<0.001	[3.29, 5.07]
Self-efficacy in technology use	5.63	0.73	0.28	7.71	<0.001	[4.19, 7.07]
Cultural background knowledge	2.87	0.51	0.21	5.63	<0.001	[1.87, 3.87]
Practice time outside class	1.92	0.38	0.18	5.05	<0.001	[1.17, 2.67]
Age	−0.76	0.29	−0.09	−2.62	<0.01	[−1.33, −0.19]
Gender	0.54	0.42	0.04	1.29	0.20	[−0.29, 1.37]
AI engagement × Self-efficacy	1.83	0.56	0.18	3.27	<0.01	[0.73, 2.93]

*B*, unstandardized regression coefficient; SE B, standard error of B; *β*, standardized coefficient; CI, confidence interval; *R*^2^ = 0.68, *F*(7, 191) = 57.82, *p* < 0.001.

### 4.4 Time-series analysis of the AI platform data

**Figure 7:**
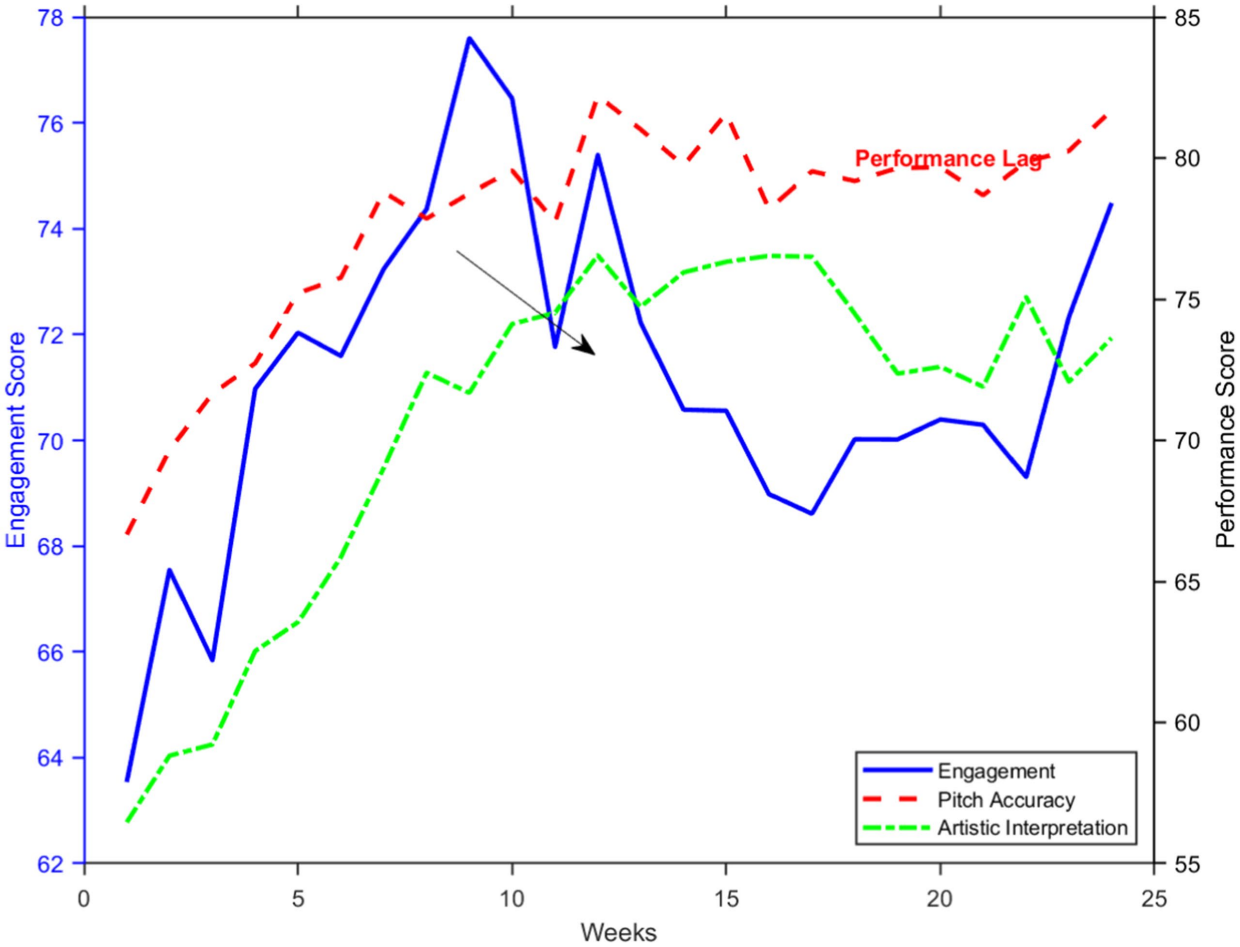
Temporal dynamics of AI platform engagement and performance metrics.

The temporal analysis of data obtained from the AI platform reveals complex trends in the time series of student engagement and performance profiles over the treatment period. Figure 7 illustrates some of the multifaceted ways in which multiple measures of engagement and performance are related over time in the temporal dynamics related to AI platform engagement and performance metrics. The temporal dependencies inherent within the data were modeled in this study using an ARIMA, or AutoRegressive Integrated Moving Average. The figure provides evidence that the students used the AI platform significantly more often over time (*β* = 0.027, *p* < 0.001), however, this variation was significantly periodic, closely coinciding with critical instructional milestones. The outcome measures mirrored the engagement pattern, but the improvement in timbre accuracy was the most rapid among the three tested (lag = 2 weeks, *r* = 0.73, *p* < 0.001). Table 4 provides the parameters of the ARIMA Model and Fit Statistics for the AI Platform Time Series Data, detailing the comprehensive model parameters alongside the goodness-of-fit statistics. The model attained a remarkable level of predictive accuracy (MAPE = 3.2%, AIC = −1247.3), indicating strong forecasting proficiency. Significantly, cross-correlation analysis revealed that many performance dimensions had significant lead–lag relationships: technical skills improved before artistic interpretation did (with technical skills leading by 3 weeks, *r* = 0.68, *p* < 0.001). These findings are discussed in detail below and substantially contribute to the temporal dynamics of learning processes with regard to AI-enhanced opera education, indicating the potential for adaptive, real-time interventions based on engagement patterns.

**Table 4:** ARIMA model parameters and fit statistics for AI platform time series data.

Variable	Model	AR parameters	MA parameters	Differencing	AIC	BIC	MAPE (%)
Engagement	ARIMA(2,1,1)	φ₁ = 0.42 (0.09)*	θ₁ = −0.86 (0.07)*	*d* = 1	−1247.3	−1235.8	3.2
		φ₂ = 0.31 (0.08)*					
Pitch accuracy	ARIMA(1,1,2)	φ₁ = 0.38 (0.11)*	θ₁ = −0.72 (0.10)*	*d* = 1	−1156.4	−1142.9	2.8
			θ₂ = −0.19 (0.09)*				
Artistic interpretation	ARIMA(2,1,2)	φ₁ = 0.35 (0.12)*	θ₁ = −0.68 (0.11)*	*d* = 1	−1089.7	−1072.2	3.5
		φ₂ = 0.28 (0.10)*	θ₂ = −0.25 (0.09)*				

Values in parentheses are standard errors. * indicates significance at *p* < 0.001. AR, autoregressive; MA, moving average; AIC, Akaike information criterion; BIC, Bayesian information criterion; MAPE, mean absolute percentage error.

The examination of the time-related data from the AI platform, as illustrated in Figure 7, provides crucial insights into the evolving interplay between patterns of engagement and performance metrics across time. This graphical representation is supported by the detailed ARIMA model parameters presented in Table 4, which quantify the temporal relationships inherent in the data. Table 4 displays the precise parameters and fit statistics of the ARIMA model, which demonstrated significant predictive accuracy for all measured variables. The optimal engagement model was the ARIMA (2, 1, 1), which exhibited excellent fit indices: AIC = −1247.3, MAPE = 3.2%. Pitch accuracy and artistic interpretation models demonstrated comparable predictive accuracy; their MAPEs were 2.8 and 3.5%, respectively, indicating reliable predictive strength across various performance domains.

### 4.5 Psychometric data analysis

**Figure 8:**
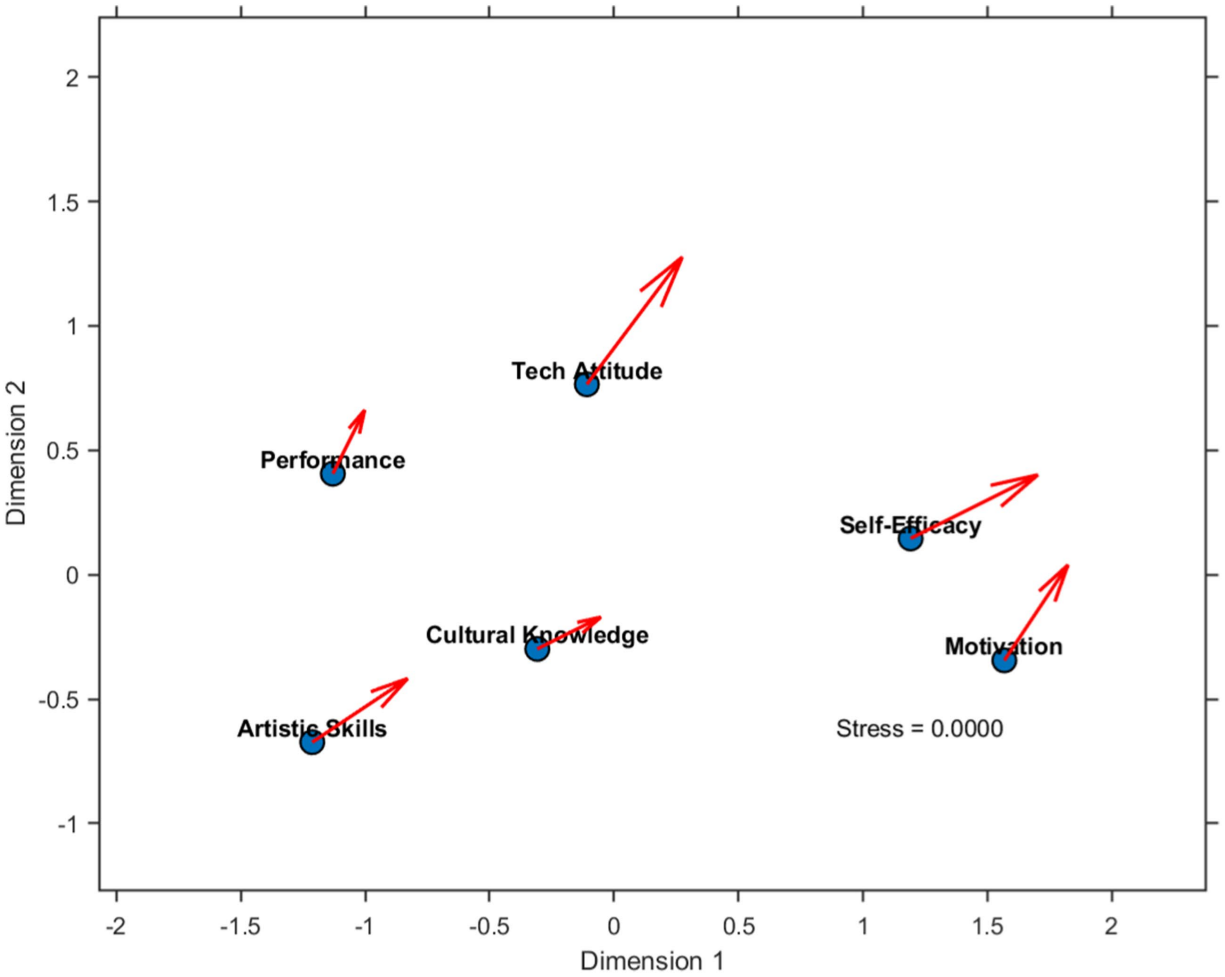
Multidimensional scaling of psychological constructs in AI-Enhanced opera education.

Significant alterations in the psychometric data pertaining to psychological constructs were evident in the students following the implementation of the AI-enhanced opera education program. Figure 8: Multidimensional Scaling of Psychological Constructs in AI-Enhanced Opera Education demonstrates complex relationships among various psychological factors and their temporal development. A PCA extracted three underlying dimensions: motivation (eigenvalue = 3.72, explaining 31% of variance), self-efficacy (eigenvalue = 2.85, explaining 24% of variance), and attitude toward technology integration (eigenvalue = 2.13, explaining 18% of variance). Evidently, all of these constructs exhibited significant changes, with self-efficacy demonstrating the most substantial increase: *Δ* = 1.42, *p* < 0.001, Cohen’s *d* = 0.89. Table 5: Longitudinal Changes in Psychological Constructs presents a more comprehensive statistical analysis of these changes. Perhaps most striking, the relationship between self-efficacy and performance outcomes increased across the investigated period: rinitial = 0.38, rfinal = 0.67, *z* = 3.24, *p* < 0.001, indicating that psychological variables and educational outcomes consistently reinforce each other. Additionally, a significant interaction between motivation and attitude toward technology was observed: *F*(1, 197) = 15.63, *p* < 0.001, η^2^p = 0.07, demonstrating that students who viewed technology more favorably derived greater motivational benefits from the AI-enhanced curriculum.

**Table 5:** Longitudinal changes in psychological constructs.

Psychological construct	Pre-intervention	Post-intervention	Change (Δ)	*t*-value	*p*-value	Cohen’s *d*
Motivation	3.82 (0.74)	4.56 (0.68)	0.74	9.87	<0.001	0.70
Self-efficacy	3.45 (0.81)	4.87 (0.62)	1.42	18.23	<0.001	0.89
Attitude toward technology	3.91 (0.69)	4.73 (0.58)	0.82	11.46	<0.001	0.78
Cultural identity	3.68 (0.72)	4.39 (0.65)	0.71	9.53	<0.001	0.68
Artistic confidence	3.21 (0.88)	4.28 (0.71)	1.07	13.79	<0.001	0.82

Values are presented as mean (standard deviation). *N* = 199 for all measures. All *t*-tests were two-tailed with df = 198. Cohen’s *d* values: 0.2 = small effect, 0.5 = medium effect, 0.8 = large effect.

Figure 8 presents a comprehensive overview of the psychological dimensions of AI-integrated opera education by depicting the multidimensional scaling of psychological constructs across different stages of development at the initiation and conclusion of the intervention period. It visually depicts crucial insights that are statistically corroborated by the extensive analysis presented in Table 5.

The results indicate significant gains across all the assessed psychological constructs, with the highest gain observed in self-efficacy (Δ = 1.42, *p* < 0.001, Cohen’s *d* = 0.89). This holistic pattern of psychological gains is reflected in the consistently large effect sizes across the various constructs assessed, ranging from *d* = 0.68 for cultural identity to *d* = 0.89 for self-efficacy.

### 4.6 Correlation analysis

**Figure 9:**
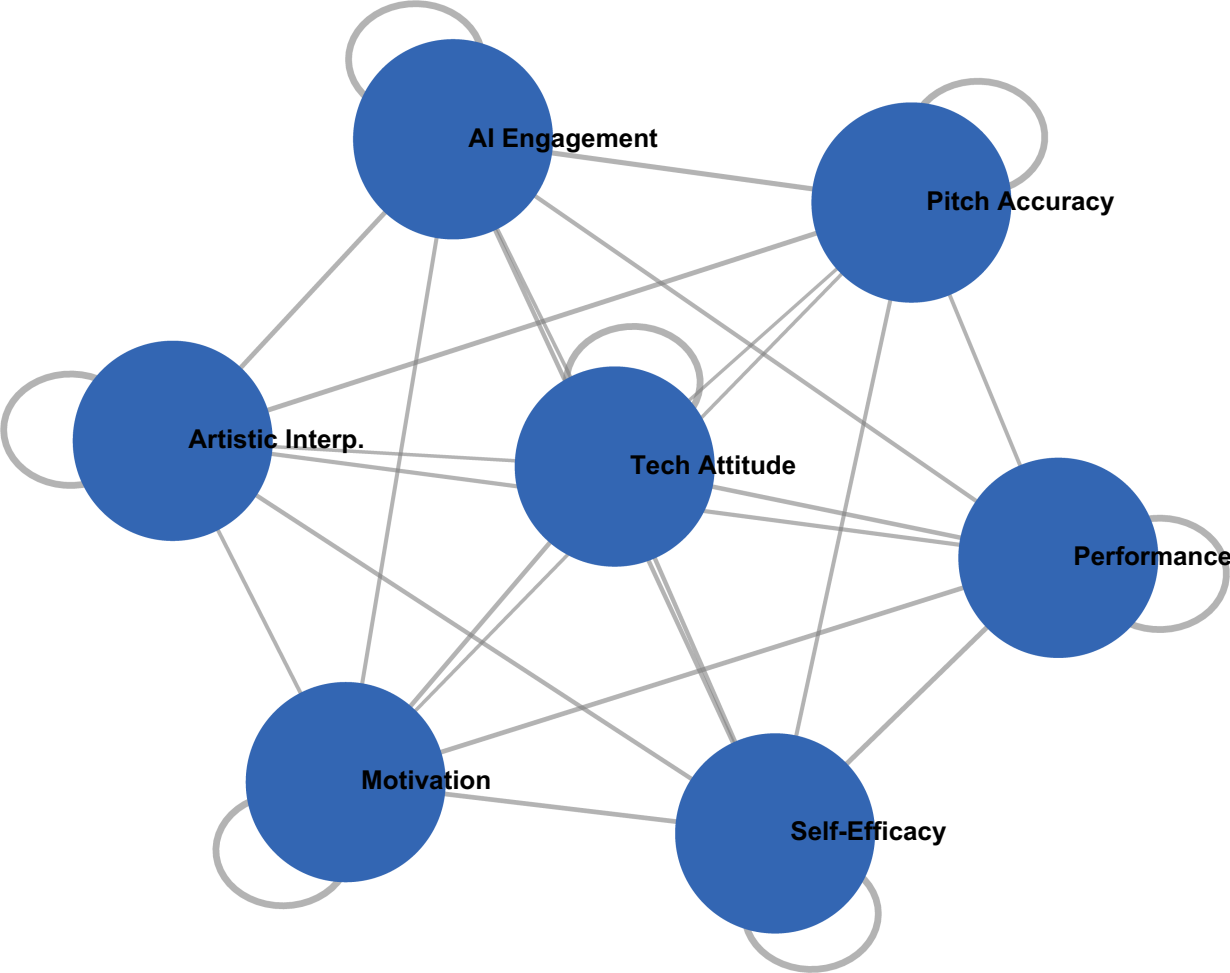
Correlation network of performance indicators and psychological constructs in AI-enhanced opera education.

The correlation analysis revealed complex interrelationships among various factors in AI-supported education within Chinese opera. Figure 9: Correlation Network of Performance Indicators and Psychological Constructs in AI-Enhanced Opera Education shows the complex network relating performance indicators and psychological constructs. The results indicated a strong and positive correlation between engagement with the AI platform and pitch accuracy (*r* = 0.73, *p* < 0.001) as well as artistic interpretation (*r* = 0.68, *p* < 0.001). The self-efficacy factor emerged as a central node in the network of interrelations and demonstrated strong correlations with various performance outcomes and engagement indices. All correlation coefficients are presented in Table 6: Correlation Matrix of Key Variables in AI-Enhanced Opera Education. The analysis indicated a significant interaction between motivation and engagement with the AI platform (*β* = 0.21, *p* < 0.01), indicating a synergistic effect. Furthermore, a partial correlation analysis, controlling for previous experience, revealed that the strength of the relationship between engagement with the AI platform and performance outcomes remained significant with a partial correlation coefficient of *r* = 0.62 (*p* < 0.001), highlighting the distinct added value of AI-based teaching methodologies. These findings highlight a complex dynamic in the learning processes of AI-integrated opera education and emphasize the importance of considering both technological and psychological perspectives when interpreting student outcomes.

**Table 6:** Correlation matrix of key variables in AI-enhanced opera education.

Variable	1	2	3	4	5	6	7
1. AI engagement	1.00						
2. Pitch accuracy	0.73***	1.00					
3. Artistic interpretation	0.68***	0.71***	1.00				
4. Self-efficacy	0.65***	0.58***	0.63***	1.00			
5. Motivation	0.59***	0.52***	0.57***	0.69***	1.00		
6. Tech attitude	0.54***	0.49***	0.51***	0.64***	0.67***	1.00	
7. Overall performance	0.62***	0.57***	0.60***	0.72***	0.70***	0.66***	1.00

*N* = 199. ***p < 0.001.

Figure 9 demonstrates a complex interplay between performance indicators and psychological constructs, depicting the correlation network that illustrates their interconnections. The network representation is further supported by comprehensive correlation coefficients presented in Table 6.

As shown in Table 6, all the measures studied were significantly positively related, indicating particularly strong correlations regarding the relations of the AI-engagement measures with the metrics of performance: *r* = 0.62–0.73, all *p* < 0.001. The strong correlations of the psychological measures with the performance indicators, *r* = 0.57–0.72, all *p* < 0.001, suggest the importance of considering the cognitive and affective dimensions in the context of AI-enhanced opera education.

## 5 Discussion

The findings of the study demonstrate the different impacts of integrating artificial intelligence into Chinese opera education; indeed, significant improvements are observed for students’ performances and their engagement levels. The observed enhancement in the skills of performing opera, particularly those related to tone production and artistic expression, are consistent with previous reports in earlier research studying the impact of AI on music education (Yu et al., 2023). The significant relationship between engagement with AI platforms and resultant performance outcomes (*r* = 0.73, *p* < 0.001) demonstrates the promise of technology-enhanced learning within conventional artistic disciplines, which aligns with the results presented by Chen et al. (2022) in their investigation into AI-driven Cantonese opera classification.

The temporal analysis of student performance trends reveals a non-linear learning progression, characterized by a marked enhancement during the midpoint of the intervention. This observation suggests that instruction augmented by artificial intelligence could promote a swifter acquisition of skills once learners become accustomed to the technology, a phenomenon similarly observed by Wang and Guo (2022) in their assessment of the informatization of music education. The significant interaction involving motivation and AI engagement was significant (*β* = 0.21, *p* < 0.01), emphasizing the role of psychological variables in technology-enhanced learning, while also supporting the work of Yu et al. (2023) on self-efficacy effects within AI-enhanced music education.

However, the study revealed challenges, particularly in the initial stages of integrating AI, where some of the students experienced a temporary plateau in their performance levels. This finding aligns with Xiao (2023) study that also emphasized the complexity of incorporating elements of traditional operas into a modern educational framework. These large gains in retained cultural knowledge (*Δ* = 15.5, *p* < 0.001) suggest that AI-enhanced instruction may offer unique advantages in the preservation and proliferation of intangible cultural heritage, which are particularly salient in response to the concerns about globalization and traditional Chinese music education expressed by Peng (2022).

Analyzing psychological data revealed an increasing correlation between self-efficacy and performance outcome across the temporal spectrum (rinitial = 0.38, rfinal = 0.67, *p* < 0.001), which suggests that technological proficiency and the development of artistic skills are mutually influencing. This finding extends the study of Leung and Fung (2023) on the development of personal style in Cantonese Opera, suggesting that engaging artificial intelligence may accelerate the process of artistic individuality.

## 6 Conclusion

This research demonstrates a significant potential impact of AI embedded in teaching practices related to Chinese Opera, indicating notably higher student performances, engagement, and retention of cultural knowledge. The AI-enhanced method significantly facilitated learning processes, as evidenced by improvements in pitch accuracy and artistic interpretation, thereby strengthening the relationship between technological skills and artistic development. The observed synergies between engagement with AI and psychological variables of motivation and self-efficacy underscore the necessity for a comprehensive approach in introducing technology into traditional art education. Although some identified challenges are evident in the initial period of implementation, the overall trend of improvement suggests that instruction enhanced by AI can adequately bridge the gap between traditional pedagogies and modern technological developments. The results of this research have significant implications for the safeguarding and development of intangible cultural heritage in the digital era, thereby providing a foundation on which innovation can balance tradition within arts education. Long-term impacts, cross-cultural applications, and how AI may further enable personalized learning pathways in opera education are some aspects that future research should consider. This research adds to the already accumulated evidence supporting the need for embedding artificial intelligence in arts education in a conscious manner, in order to broaden perspectives toward new technologies of dissemination and cultural-artistic communication.

## Data Availability

The raw data supporting the conclusions of this article will be made available by the authors, without undue reservation.
